# Role of Factor Xa Inhibitors in Cancer-Associated Thrombosis: Any New Data?

**DOI:** 10.1155/2011/196135

**Published:** 2011-10-15

**Authors:** Ali Zalpour, Michael H. Kroll, Vahid Afshar-Kharghan, Syed Wamique Yusuf, Carmen Escalante

**Affiliations:** ^1^Pharmacy Clinical Programs, Division of Pharmacy, The University of Texas MD Anderson Cancer Center, Houston, TX 77030, USA; ^2^Section of Benign Hematology, The University of Texas MD Anderson Cancer Center, Houston, TX 77030, USA; ^3^Department of Cardiology, The University of Texas MD Anderson Cancer Center, Houston, TX 77030, USA; ^4^Department of General Internal Medicine, The University of Texas MD Anderson Cancer Center, Houston, TX 77030, USA

## Abstract

The association between cancer and venous thromboembolism (VTE) has been well documented in the literature. Prevention and treatment of VTE in cancer patients is imperative. Typically, the mainstay regimen for VTE prevention and treatment has been anticoagulation therapy, unless contraindicated. This therapy consists of unfractionated heparin (UFH), low-molecular-weight heparin (LMWH), factor Xa inhibitor, or vitamin K antagonist (VKA). Current guidelines recommend LMWH over VKA for the treatment of VTE in cancer patients. Factor-specific anticoagulants have been proven safe and effective, and recently factor Xa inhibitors have emerged as a treatment alternative to heparins and VKA. Currently, three factor Xa inhibitors have been identified: fondaparinux (the only one approved so far by the US Food and Drug Administration), idraparinux (in clinical trials), and idrabiotaparinux (in clinical trials). This paper will examine the role of these agents, focusing on fondaparinux, for the prevention and treatment of VTE in cancer patients.

## 1. Introduction

The association between cancer and venous thromboembolism (VTE) has been well recognized and established [1]. Cancer patients have a 4-fold higher risk of developing VTE than do patients without cancer, and chemotherapy increases that risk to 6-fold [2]. In cancer patients undergoing surgical procedures, rates of postoperative VTE can increase 2-fold greater than rates of postoperative VTE in patients without cancer [3]. Frequency of VTE has increased by up to 28% in years 1995 to 2003 in hospitalized cancer patients and with the higher mortality rates compared to those hospitalized cancer patients without VTE (16.3% versus 6.3%, *P* < 0.0001) [4]. 

Given that the 1-year survival rate in cancer patients with VTE is much lower than in cancer patients without VTE (12% versus 36%), appropriate and effective thromboprophylaxis—both pharmacologic and nonpharmacologic—is imperative [9]. Effective thromboprophylaxis can minimize mortality and morbidity, potentially affect survival, and lower health-care costs associated with VTE. 

The National Comprehensive Cancer Network (NCCN), the American Society of Clinical Oncology (ASCO), and recently the American College of Chest Physicians (ACCP) have published guidelines for the prevention and treatment of VTE in cancer patients (Table 1). These guidelines recommend using unfractionated heparin (UFH), low-molecular-weight heparins (LMWHs), and, recently, direct factor Xa inhibitors for the prevention of VTE in cancer patients who are hospitalized [5–8]. 

Currently, prophylaxis for catheter-related thrombosis is not recommended owing to conflicting evidence and a lack of major clinical trials [10]. For treatment of VTE in cancer patients, guidelines recommend LMWH as first-line therapy and vitamin K antagonist (VKA) as second-line therapy. The recommended duration of treatment ranges from 3 to 12 months, depending on which set of guidelines is followed. For long-term treatment of VTE in cancer patients, guidelines do not address the role of factor Xa inhibitors [5–8].

Factor-specific anticoagulants have been proven safe and effective, and, recently, factor Xa inhibitors have emerged as an alternative therapy for VTE in cancer patients. In this paper, we examine the role of injectable factor Xa inhibitors in cancer patients, focusing on fondaparinux, the only such agent yet approved by the US Food and Drug Administration (FDA). The paper covers clinical pharmacology, clinical pharmacokinetics, and available data from clinical trials in cancer patients. We also addressed the safety of using factor Xa inhibitors for the prevention and long-term treatment of VTE in special populations of cancer patients, such as patients who are obese, have renal insufficiency, or have a high risk of bleeding. Finally, we presented recent and emerging data on idraparinux and idrabiotaparinux, two factor Xa inhibitors currently being investigated in clinical trials for the prevention and treatment of VTE in cancer patients. 

## 2. Fondaparinux

### 2.1. Pharmacodynamic Profile of Indirect Factor Xa Inhibitor

**Figure 1:**
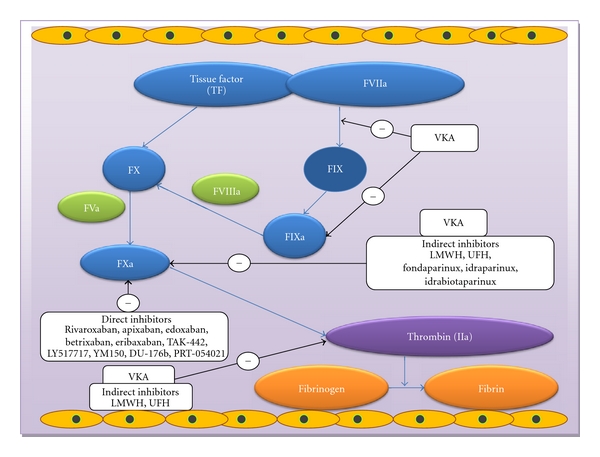
Mechanism of action of anticoagulants. Abbreviations: vitamin k antagonist (VKA); low-molecular-weight heparin (LMWH); unfractioned heparin (UFH).

After activation of the coagulation cascade by either the intrinsic or extrinsic pathways, the generation of factor Xa is amplified. Factor Xa is the first step in the common pathway that leads to the generation of thrombin which converts prothrombin to thrombin and finally the formation of the fibrin (Figure 1) [11]. Briefly, VKAs inhibit the formation of factors II, VII, IX, X and the natural anticoagulant proteins C and S [12]. UFH and LMWHs have more specificity for inhibition of coagulation factors such as factor Xa and factor IIa. LMWH has been shown to bind mostly to factor Xa and minimally to factor IIa through activation of antithrombin (AT) III (ATIII) [12]. 

In recent years, more targeted anticoagulants such as fondaparinux (1728 Da or pentasaccharides) have emerged that specifically target factor Xa. Fondaparinux does not inhibit factor IIa since more than18 monosaccharides are required for simultaneous binding to AT and factor IIa [13]. Fondaparinux binds reversibly to AT in a 1 : 1 ratio through a noncovalent bond, which causes conformational change and then selectively and rapidly inhibits factor Xa and thereby inhibition of thrombin (inhibition of 1 unit of factor Xa results in *∼*50 units lower concentration of thrombin). Fondaparinux binding to AT causes an approximate 300-fold increase in the rate at which AT inhibits factor Xa [14, 15]. Direct factor Xa inhibitors inhibit free factor Xa and bound factor Xa in the prothrombinase complex, whereas indirect factor Xa (fondaparinux) inhibitors target factor Xa through AT. Factor Xa inhibitors can block the formation of thrombin rather than the inhibition of thrombin activity [16]. 

Currently, there are three injectable indirect factor Xa inhibitors in various stages of development: fondaparinux, idraparinux, and idrabiotaparinux (Table 2). Only fondaparinux has been approved by the FDA for the prevention and treatment of VTE [17, 18]. 

Presently, there are oral formulations of direct factor Xa inhibitors, but these are beyond the scope of this paper; oral factor Xa inhibitors that are in various phases of clinical trials include rivaroxaban (Xarelto), which has been approved in Europe for prevention of VTE, apixaban, edoxaban, betrixaban, eribaxaban, TAK-442, LY517717, YM150, DU-176b, and PRT-054021 [19].

### 2.2. Pharmacokinetic Profile

Fondaparinux exhibits the following pharmacokinetic parameters: complete bioavailability of *∼*100%; peak plasma concentration of 0.34 mg/L (dose of 2.5 mg); time-to-peak plasma concentration of 1.7 hours; linear pharmacokinetics in the 2 mg to 8 mg dose range. Fondaparinux is excreted via the kidneys (64% to 74%) with a terminal half-life (*T*_*β*1/2_) of *∼*17 hours [20]. Fondaparinux is not metabolized by the cytochrome P450 (CYP) enzyme in the liver [15].The volume of distribution (Vd) and protein binding of fondaparinux is estimated to be from 7 L to 11 L and >97%, respectively; hence, fondaparinux does not distribute to extravascular space such as adipose tissue. Fondaparinux has a negligible nonspecific binding to *α*-_1_-acid-glycoprotein [15].

Pharmacokinetic studies have produced peak and trough levels of fondaparinux at steady state (*C*_ss max⁡/min⁡_) of 0.39 to 0.50 mcg/mL and 0.14 to 0.19 mcg/mL for the 2.5-mg dosing schedule, respectively. The peak and trough levels (or ranges) of fondaparinux at *C*_ss max⁡/min⁡_ for the 5-mg, 7.5-mg, and 10-mg dosing schedules are 1.20 to 1.26 mcg/mL and 0.46 to 0.62 mcg/mL [18].

### 2.3. Comparative Efficacy in VTE Prevention Trials

Primary data using fondaparinux for prevention of VTE in cancer patient is clearly lacking. In a study of elderly patients (number (*n*) = 849) with acute medical illnesses (*∼*15% had cancer), patients who received fondaparinux (2.5 mg per day per day for 14 days) had a relative risk reduction (RRR) of 46% (95% confidence interval (CI): 7.7% to 69.3%) in VTE compared with patients who received the placebo (5.6% versus 10.5%). The patients who received fondaparinux had a 0.4% incidence of major bleeding, and their 32-day mortality rate was 3.3%, while patients who received the placebo had a 32-day mortality rate of 6.0% (*P* = 0.006). In this study, fondaparinux provided the same efficacy across body weight ranges of 32 kg to 111 kg, and bleeding was not related to body weight [21]. 

Turpie et al. showed a VTE rate reduction of 69.8% in patients who underwent major abdominal surgery (40% of patients had surgery for cancer); patients received either fondaparinux (2.5 mg per day or prophylactic dose) plus intermittent pneumatic compression (IPC) or IPC alone, with low major bleeding rates of 1.6% bleeding rate in the fondaparinux plus IPC group and the 0.2% in the IPC alone group (*P* = 0.006) [22]. The first injection of fondaparinux was given 6 to 8 hours after surgical closure, and the second injection of fondaparinux was given 16 to 28 hours after the first injection; an epidural, if used, was removed 2 hours prior to the first injection. In this study, the efficacy of fondaparinux was proven irrespective of age, gender, weight (mean, 82 kg), or type and duration of surgery. The overall mortality rate was 1.3% in the fondaparinux plus IPC group (1 fatal pulmonary embolism (PE)) and 0.8% in the IPC group (1 fatal PE, *P* = 0.42) [22].

In another study of VTE prevention in surgery patients, Agnelli et al. evaluated a subset of cancer patients (*n* = 954) who underwent major abdominal surgery and demonstrated that rate of VTE in patients who received fondaparinux (2.5 mg per day) was 4.7% whereas the rate of VTE in patients who received dalteparin (5000 units per day) was 7.7%; the RRR was 38.6 % (95% CI: 6.7% to 59.7%), and the incidence rate of major bleeding was 3.4% versus 2.5% (*P* = 0.355) [23]. Major bleeding occurred in 2.8% of patients who received their first fondaparinux injection at least 6 hours after surgery closure and in 3.4% of patients who received their first fondaparinux dose within 6 hours of surgery closure [23].

Overall, these studies suggest that fondaparinux could be an option for prevention of VTE in cancer patients who are hospitalized for either an acute medical illness or a surgical procedure.

### 2.4. Comparative Efficacy in VTE Treatment Trials

Primary data of fondaparinux for treatment of VTE cancer patients is also lacking. Two studies have shown the similar efficacy of fondaparinux versus LMWH and VKA for the initial phase of VTE treatment that enrolled 10% of patients with cancer [24, 25]. A subgroup analysis of cancer patients in the Matisse-DVT trial showed that recurrent VTE rates in the initial treatment period, for the entire study period, and in patients with advanced cancer were as follows for the fondaparinux group versus the enoxaparin group: 2.4% versus 0.0%, for an absolute difference of 2.4% (95% CI: −0.3% to 5.0%, *P* = 0.080); 12.7% versus 5.4%, for an absolute difference of 7.3% (95% CI: 0.1% to 14.5%, *P* = 0.046); 11.5% versus 3.7%, for an absolute difference of 7.8% (95% CI: −6.4% to 22.0%, *P* = 0.28). Major bleeding rates in cancer patients during the entire study period were 7.1% in the fondaparinux group versus 7.2% in the enoxaparin group, for an absolute difference of −0.1% (95% CI: −6.7% to 6.5%, *P* = 0.99). Mortality rates were 18.3% in the fondaparinux group versus 15.3% in the enoxaparin group, for an absolute difference of 2.9% (95% CI: 6.6% to 12.4%). This subgroup analysis of cancer patients showed that during the entire study period there was a trend toward higher rates of recurrent VTE in fondaparinux-treated patients (12.7% in the fondaparinux group versus 5.4% in the enoxaparin group; *P* = 0.046) [26]. One possible explanation for the higher rates of recurrent VTE in the fondaparinux group could be that 8.7% of the fondaparinux-treated patients (bridged with VKA) had an international normalized ratio (INR) <2.0 (at the time of recurrent DVT versus 1.8% of the enoxaparin-treated patients; 3.6% of patients in the enoxaparin group had therapeutic INRs (2.0 to 3.0) versus 1.6% of patients in the fondaparinux group [27]. 

A subgroup analysis of cancer patients in the Matisse-PE study reported that recurrent VTEs in the initial treatment period, for the entire study period, and in patients with advanced cancer were as follows for the fondaparinux group versus the UFH group: 0.9% versus 3.9%, for an absolute difference of −3.0% (95% CI: −6.8% to 0.8%, **P** = 0.12); 8.9% versus 17.4%, for an absolute difference of −8.3% (95% CI: −16.7% to 0.1%, **P** = 0.054); 16.0% versus 29.0%, for an absolute difference of −13.0% (95% CI: −35.0% to 8.5%, **P** = 0.24), respectively. Major bleeding rates in cancer patients during the entire period were 3.6% in the fondaparinux group versus 6.3% in the UFH group, for an absolute difference of −2.7% (95% CI: −8.1% to 2.7%, **P** = 0.33). Mortality rates during the 3-month followup were 25.0% in the fondaparinux group versus 18.8% in the UFH group, for an absolute difference of 6.2% (95% CI: −4.2% to 16.7%). In this subgroup analysis, higher rates of recurrent VTE during the entire study were observed in the UFH group compared with the fondaparinux group (17.2% versus 8.9%, **P** = 0.054). Patients randomized to receive UFH plus VKA had lower INR rates at the time of VTE recurrence than did patients in the fondaparinux group, 7.0% and 2.7%, respectively, for an absolute difference of 0.4% (95% CI: −10.0% to 1.0%) [27].

### 2.5. Dosing of Fondaparinux

#### 2.5.1. Dosing of Fondaparinux in Underweight and Overweight Patients with Cancer

Fondaparinux is dosed according to the patient's body weight based on the following schema: for a body weight of <50 kg, the dose is 5 mg subcutaneously (SQ) daily; for a body weight of 50 kg to 100 kg, the dose is 7.5 mg SQ daily; for a body weight >100 kg, the dose is 10 mg SQ daily [18]. 

 Pooled analysis of the effect of obesity (cutoff, 30 kg/m^2^) on outcomes in the Matisse trials (Matisse-DVT and Matisse-PE) has been reported. Body weight (kg) and body mass index (BMI; kg/m^2^) ranges reported in this analysis were from 33 kg to 216 kg and from 12.8 kg/m^2^ to 8.1 kg/m^2^, respectively [28]. When investigators analyzed the data from the Matisse trials, they observed no difference in recurrent VTE rates between fondaparinux and heparins (enoxaparin or UFH) across body weight and BMI categories as follows: body weight ≤100 kg (3.90% versus 4.45%, *P* = 0.42); body weight >100 kg (4.0% versus 5.7%, *P* = 0.41); BMI <30 kg/m^2^ (3.9% versus 4.5%, *P* = 0.42); BMI ≥30 kg/m^2^ (3.7% versus 4.8%, *P* = 0.40). Bleeding rates did not differ between fondaparinux and heparins (enoxaparin or UFH): body weight ≤100 kg (1.3% versus 1.2%, *P* = 0.77); body weight >100 kg (0.4% versus 0.8%, *P* = 0.62); BMI <30 kg/m^2^ (1.5% versus 1.2%, *P* = 0.53); BMI ≥30 kg/m^2^ (0.3% versus 1.1%) [28]. The results of this study showed that the safety and efficacy of fondaparinux for initial treatment of VTE was comparable to heparin across different body weight and BMI categories [28]. In the Matisse trials, cancer patients comprised 25% of total patients enrolled overall [24, 25]. Until further evidence is available in cancer patients with extremes of body weight and BMI, data presented by the pool analysis should be interpreted with caution. 

#### 2.5.2. Dosing of Fondaparinux in Cancer Patients with Renal Disease

Fondaparinux is excreted via the kidneys, is contraindicated in patients with CrCl <30 mL/min, and should be used with caution in patients with CrCl from 30 to 50 mL/min [18]. Close attention to estimation of the glomerular filtration rate (GFR) is imperative in dosing of antithrombotics such as fondaparinux that has a long *t*_1/2_. 

 Estimation of the GFR calculated with the Cockcroft-Gault (CG) formula differs widely from calculations made with the Modification of Diet in Renal Disease (MDRD) formula in low BMIs and in patients older than 75 years [29]. Another study reported higher rates of bleeding and transfusions in patient with acute coronary syndrome that were prescribed unadjusted doses of antithrombotics based on CG [30]. 

The CG formula may better assess GFR in patients with normal serum creatinine (Scr) levels who are at risk of developing kidney disease such as patients who have diabetes, who have stage 1 or 2 CKD, or who are elderly. The MDRD formula may be more appropriate for predicting the GFR in patients with CKD who do not have normal Scr levels, who have a GFR <30 mL/min, and who have a high BMI. Neither CG nor MDRD was accurate in the following patients: heart transplant patients, kidney donors, patients with advanced liver disease, and hospitalized patients [26]. 

In a pharmacokinetic simulation study, Turpie et al. showed that dosing fondaparinux at 1.5 mg and 2.5 mg SQ daily provided comparable trough plasma concentration (*C*_min⁡_), peak plasma concentration (*C*_max⁡_), and area under the curve (AUC_0–24 h_) at days 1, 7, and 28 in patients with a CrCl of ≥20 mL/min, <50 mL/min, and CrCl >50 mL/min. However, dosing fondaparinux at 2.5 mg produced much higher *C*_min⁡_, *C*_max⁡_, and AUC_0–24 h_  in patients with 20 mL/min ≥ CrCl < 50 mL/min [31]. The fondaparinux 1.5-mg dosing schedule may be a safe and effective alternative to 2.5-mg recommended dose for the prevention of VTE; however, determining the most effective dosing schedule for fondaparinux requires further trials and investigation.

 Results from studies of fondaparinux (2.5 mg) used as an anticoagulant during hemodialysis have shown the efficacy of fondaparinux in preventing circuit clotting during dialysis; however, anti-Xa activity is increased and may potentially increase the risk of bleeding in such patients on dialysis [32, 33]. 

To our knowledge, there have been no studies assessing the safety and efficacy of fondaparinux in patients with end-stage renal disease and concomitant VTE. Dialysis patients with an acute medical illness who are hospitalized should receive prophylaxis with UFH [34]. 

### 2.6. Monitoring Anti-Xa Levels in Patients Receiving Fondaparinux

Currently, to our knowledge, there have been no clinical trials to validate peaks and troughs in the efficacy of fondaparinux. 

Fondaparinux-specific anti-Xa assay should be used to monitor the anti-Xa levels of fondaparinux in patients who have unstable renal function, are elderly, are morbidly obese, or have a low BMI [35]. Activated partial thromboplastin time (aPTT) and prothrombin time (PT) cannot be used to measure anticoagulant properties of fondaparinux because these test systems measure the inhibition of thrombin and not factor Xa [36]. Fondaparinux has been shown to affect the protein S assay in vitro, resulting in false elevation of protein S levels; however, the significance of these findings is yet to be determined [37]. Linkins et al. have shown that fondaparinux has no effect on aPTT or activated clotting time (ACT), contrary to heparins; therefore, such tests are insensitive to the anticoagulant properties of fondaparinux [38]. Smogorzewska et al. showed that fondaparinux (prophylactic or therapeutic dose) prolonged PT by 1 second and the aPTT by 4 to 5 seconds without significant changes in fibrinogen, AT, and thrombin time assays [39]. Depasse et al. showed that LMWH anti-Xa (IU/mL) levels varied significantly between different assays for the same fondaparinux concentrations (*P* < 0.01) [40]. Methodology of determining factor Xa activity of fondaparinux requires a chromogenic assay that is calibrated to fondaparinux but not to LMWH or UFH. 

### 2.7. The Role of Fondaparinux in Critically Ill Cancer Patients

Despite prophylaxis use in the intensive care unit (ICU), the rate of DVT is about 5.1% to 5.8% and the rate of PE is about 1.2% to 2.3%, the major bleeding rate is approximately 13.0%, and the mortality rate is 15.0% [41]. The pharmacokinetic properties of drugs in ICU patients may be altered owing to reduced hepatic metabolism, reduced transport function, intestinal atrophy, increased or decreased extravascular fluids, reduced CrCl, altered tissue permeability, or decreased protein binding of the drugs [42]. 

Administration of vasopressors to patients who received fondaparinux (2.5 mg) did not change the mean range of anti-Xa levels compared with those without vasopressors (0.2 to 0.4 IU/mL versus 0.3 to 0.2 IU/mL, *P* = 0.06). Interestingly, investigators found no occurrence VTE and 2 minor bleeds with anti-Xa of 0.24 and 0.44 IU/mL [43]. 

There are currently no guidelines for dose adjustment of fondaparinux based on anti-Xa levels, and, to our knowledge, no studies have compared the effectiveness of fondaparinux versus heparin in ICU patients. Use of pharmacological agents for DVT prophylaxis in ICU patients should be based on evidence, the half-life of the agent, and the availability of an antidote if bleeding occurs.

### 2.8. Role of Fondaparinux in Cancer Patients with Heparin-Induced Thrombocytopenia

Heparin-induced thrombocytopenia (HIT) is an immune-mediated disorder caused by immunoglobulin G (IgG) antibodies that bind to platelet factor 4 (PF4), resulting in the generation and release of procoagulant microparticles such as thrombin [44]. Thrombin causes disseminated arterial and venous thrombi and has a reported absolute risk of thrombosis of 35% to 57% in some clinical settings [45]. Mortality rates in patients with HIT and thrombosis can be as high as 30%, and 20% of patients who survive require a limb amputation [46]. 

Patients who can carry risk of HIT of >1% are surgical patients who received UFH (prophylactic dose or treatment dose) for more than 4 days. Patients who carry risk of HIT of 0.1% to 1% are (1) medical patients who received UFH (prophylactic dose or therapeutic dose) for more than 4 days; (2) surgical patients who received LMWH for more than 4 days or heparin flushes for more than 4 days; (3) medical or surgical patients who were switched from UFH to LMWH carry an estimated risk HIT of 0.1% to 1.0%. Finally, medical or surgical patients who received LMWH for more than 4 days, medical patients received heparin flushes only, or any patients that have who received UFH or LMWH for fewer than 4 days have a risk of HIT of 0.1% [47]. 

Fondaparinux is considered a pentasaccharide and has an average molecular weight of 1.7 kDa and only 5 saccharide residue. Evidence suggests that an increased risk for HIT-related antigen production is dependent on the molecular weight and length of the polysaccharides (>2.4 kDa and >10 saccharide units). Typically, in LMWHs, the range of molecular weight and saccharide residues vary from 4.5 kDa to 7.5 kDa and 13 to 15 saccharide residues, respectively. In UFH, the molecular weight and saccharide residues are much higher, 15 kDa and 45 kDa. It has been hypothesized that the lower saccharide residue, the lower antigenicity [48]. 

Testing the sera of patients from 2 clinical trials, investigators showed that the PF4-fondaparinux complex, but not the PF4-LMWH complex, is readily recognized by antibodies generated during HIT and that the risk of HIT associated with fondaparinux is very low [49]. Kovacs reported a successful case series of five patients with the diagnosis of HIT who were treated with 7.5 mg of fondaparinux [50]. In this case series, time to platelet recovery was between 2 and 9 days, with no new thrombotic activity or major bleeding [50].

Wester et al., in a retrospective study of seven critically ill patients with suspected HIT, investigated the role of fondaparinux at a dosing schedule of 2.5 mg per day, with dose adjustments according to renal function (adjusted to 1.5 mg) [51]. HIT antibodies were absent in all patients; five patients were classified as unlikely to have HIT, whereas two patients were classified as possibly having HIT. During treatment with fondaparinux, platelets recovered in five patients but did not recover in the other two. The authors concluded that the role of fondaparinux in cases of suspected HIT might be as bridging until HIT is confirmed [51].

Several case reports in the literature have advocated the use of a therapeutic dose of fondaparinux in the management of HIT [52]. Lobo et al. showed in a prospective pilot trial comparing HIT patients treated with fondaparinux (*n* = 7) with historical controls treated with direct thrombin inhibitor (DTI; *n* = 10) that 7 of 7 fondaparinux-treated patients had complete platelet recovery time (increase from baseline by at least 30% of nadir to >100,000/mm^3^ by day 7) and no fondaparinux-treated patients experienced new thrombotic events, major bleeding, or death by day 30. In the historical control group, 8 of 10 patients achieved platelet recovery with no new thrombotic events or major bleeding, but 4 had limb gangrene that might have been due to inappropriate dosing of warfarin in relation to the diagnosis of HIT [53].

NCCN guidelines considers use of fondaparinux for treatment of HIT as unlabeled use, and the ACCP suggests that after recovery of platelet levels during initial treatment with DTI, the DTI can be replaced with fondaparinux in therapeutic doses and bridging to warfarin [5, 47].

The use of fondaparinux in patients with HIT has been challenged. In 2003, Warkentin et al. reported a platelet serotonin-release assay of 90% (normal value, <20%) and an anti-PF4 polyanion enzyme immunoassay of 1.871 units (normal value, <0.40) in a patient who was not predisposed to heparin and was postoperatively on fondaparinux (2.5-mg dosing schedule), with a concomitant platelet drop and signs of thrombosis. The authors indicated that in rare cases fondaparinux can activate antibodies against PF4 that resemble the antibodies in HIT [54].

 Rota et al. reported a strong optical density (OD) of 1.700 units without thrombosis in a patient exposed to fondaparinux after undergoing a total hip replacement. Three years earlier, the patient had a history of HIT with thrombosis due to nadroparin. The authors concluded that fondaparinux should be used with caution in patients with HIT [55].

 Recently, Alsaleh et al. also reported an OD of 1.75 units (normal value, <0.45 U), heparin-dependent platelet activation at a dilution of 1 in 8 (3% release at 0 U/mL, 100% at 0.1 U/mL, and 0% at 100 U/mL UFH), with new DVT and concomitant thrombocytopenia in a patient on fondaparinux prophylaxis following a surgical procedure. The patient had been on prophylaxis with LMWH postoperatively for 3 days. In this case, platelet activation was also observed with pharmacological doses of enoxaparin (0.1 U/mL), but not in the presence of fondaparinux [56]. Treatment of cancer patients with HIT should not differ from treatment of patients who do not have cancer, and treatment consists of DTIs (such as lepirudin, bivalirudin, or argatroban) for the acute phases of HIT followed by long-term VKA after platelet recovery [57].

Until further evidence is available from randomized clinical trials, fondaparinux should be used with caution to treat HIT in cancer patients.

### 2.9. Bleeding Risk Assessment

To date, there is no validated bleeding risk assessment in cancer patients; however, several bleeding assessment tools have been examined in clinical trials. Assessment of bleeding risk will enable closer monitoring of patients who are at risk of bleeding while on anticoagulation therapy. In one study, risk of bleeding associated with anticoagulation in cancer patients was about 4.2% during 90 days of therapy, with a mortality rate greater than 60% within 30 days of a major bleeding event [58]. 

Data for management of VTE in cancer patients with thrombocytopenia is lacking. Transient thrombocytopenia due to chemotherapy doubles the bleeding risk from 10% at a platelet count of 20,000/mm^3^ to 20% when the platelet count drops below 10,000/mm^3^ in solid tumor patients [59].

NCCN considers platelet counts of less than 50,000/mm^3^ as a contraindication to prophylactic or therapeutic anticoagulation therapy [5, 6]. ASCO recommends using therapeutic anticoagulation in cancer patients with preexisting thrombocytopenia with caution. Validated bleeding assessment tools in the literature are evolving. The prospective registry Registro Informatizado de La Enfermedad Thromboembólicà (RIETE) investigators have identified variables such as recent major bleed, renal dysfunction, anemia, cancer, clinically overt PE, and advanced age as being associated with major bleeding. When RIETE investigators assigned point scores for bleeding based on risk factors, the incident rate of major bleeding was 0.1% for low risk, 2.8% for intermediate risk, and 6.2% for high-risk patients. (Table 3) [60].

In a recent analysis of the prospective RIETE registry, investigators identified variables such as age >75 years, recent major bleeding, immobility ≥4 days, metastatic cancer, anemia, platelet count <100,000/mm^3^, abnormal PT, and CrCl < 30 mL/min as independent risk factors for bleeding. Then investigators composed a risk scoring system based on assigned points to each variable to predict the risk of fatal bleeding during 90 days following diagnosis of VTE. Patients with a risk score of <1.5 points had a bleeding incidence rate of 0.16%, those with a risk score of 1.5 to 4.0 had a bleeding incidence of 1.06%, and those with score of >4.0 had a incidence of 4.24% of fatal bleeding with the likelihood ratio 0.29 for the low-risk group, 1.92 for the moderate-risk group, and 7.95 for the high-risk group (Table 3) [61]. We recommend that patients with any of the following risk factors for major bleeding should be closely monitored while on anticoagulation: age (>75 years), presence of metastatic cancer, immobility, platelet count <100,000/mm^3^, anemia, recent major bleed, coagulopathy, and CrCl < 30 mL/min.

### 2.10. Reversal of Fondaparinux

As described before, the incidence of bleeding associated with fondaparinux ranges from 1.1% to 7.2% [27, 28].

Following the assessment of bleeding risk, choosing long-acting anticoagulants requires adequate reversibility by an antidote in cases of bleeding. VKAs can be reversed with administration of vitamin K, LMWH can be partially reversed with the administration of protamine, and UFH can be completely reversed with administration of protamine; in bleeding events, transfusion of red blood cells is required as well [62]. 

There is limited experience with reversal of fondaparinux in bleeding cases, and, to date, there are no studies conducted specifically in cancer patients. There are case reports and limited laboratory studies alluding to judicious use of recombinant factor VIIa (rVIIa) for reversal of bleeding associated with fondaparinux. rVIIa is an activated factor VIIa that is approved for use in cases of congenital factor VII deficiency, patients with factor VIII or IX inhibitors, and cases of Glanzmann thrombasthenia; however, in recent years, it has been used “off-label” for reversal of warfarin, central nervous system bleeding, uremia, bone marrow transplantation, type III von Willebrand disease, factor XI deficiency, Jehovah's Witness hematologic malignancies, liver disease (cirrhosis, liver transplantation, or fulminant hepatic failure), and in platelet disorders (quantitative and qualitative). rVIIa primarily binds to TF following injury to the vessel wall and activates factor X to factor Xa, leading to conversion of prothrombin to thrombin. Thrombin activates platelets, converts factor V to factor Va and factor VIII to factor VIIIa, and finally activates thrombin-activable fibrinolysis inhibitor (TAFI), which downregulates fibrinolysis. The usual dose if rVIIa is 90 *μ*g/kg administered intravenously [63].

In a randomized, placebo-controlled study of 16 healthy male subjects who received a single dose of fondaparinux (10 mg), the effect of a single dose of 90 *μ*/kg intravenous (IV) bolus of rFVIIa was studied on coagulation parameters. Thrombin generation time (TGT) was rapidly normalized by administration of rFVIIa and persisted for 6 hours (*P* < 0.001). The endogenous thrombin potential (ETP) at 2 and 8 hours was 9% higher in the fondaparinux plus rVIIa than in the fondaparinux group alone (*P* = 0.056). Prothrombin activation was increased by 34% at 2 and 8 hours in the fondaparinux plus rVIIa compared with the fondaparinux group alone (*P* = 0.022). The AUC analysis between time points 2 and 8 hours showed a significant reduction in the aPTT (*P* = 0.015). The PT was reduced 26% after the administration of rVIIa (14.3 ± 0.9 to 9.2 ± 0.9 seconds; *P* < 0.0001). The authors concluded that administration of rFVIIa as a single 90 *μ*/kg IV bolus could neutralize the anticoagulant effect of fondaparinux (10 mg) [64].

 The effect of 90 *μ*/kg IV bolus of rVIIa on clot lysis time in 8 healthy volunteers when added to 10 mg of fondaparinux was also studied by Lisman et al. In this study, the authors found a decrease in the clot lysis time on addition of fondaparinux (control 80 ± 13 minutes, fondaparinux 65 ± 15 minutes; mean ± SD, *P* < 0.001), and on addition of rVIIa to fondaparinux-anticoagulated plasma in this group, a significant increase in clot lysis time was observed (from 65 ± 15 minutes to 72 ± 13 minutes, *P* < 0.01), and this effect lasted for up to 8 hours. The authors concluded that rVIIa may be an alternative option for reversal of bleeding due to fondaparinux [65].

Gerotziafas and his colleagues have concluded that recombinant factor VII (rFVIIa) partially reverses the effect of fondaparinux on inhibition of thrombin generation. In their study, the effect of concentrations of fondaparinux from 0.1 to 1.0 *μ*g/mL (concentrations required for prophylaxis and treatment of VTE) and the influence of rVIIa at 1 *μ*g/mL on various parameters such as lag time (minutes), thrombin *C*_max⁡_ (nM), *T*_max⁡_ (minutes), and ETP (nM × minutes) were studied. In the presence of fondaparinux 0.5 *μ*g/mL rVIIa, both the lag time and *T*_max⁡_ of thrombin generation were decreased by 1 minute. At 0.8 *μ*g/mL to 1.0 *μ*g/mL of fondaparinux, rVIIa reduced the lag time by 3 ± 1.5 minutes and the *T*_max⁡_ of thrombin generation by 4 ± 2 minutes (*P* < 0.05). The addition of rVIIa to 0.5 *μ*g/mL or lower concentration of fondaparinux did not modify the *C*_max⁡_ and ETP, but at concentrations of 0.8 *μ*g/mL of fondaparinux, rVIIa slightly increased *C*_max⁡_ and ETP (63 ± 15 nM) and (840 ± 330 nM × minutes) (*P* > 0.05) [66].

A case of rVIIa (90 *μ*g/kg) to a 76-year-old patient who was on fondaparinux (7.5 mg) following head trauma due to falls has been described in the literature. The patient received the dose of rVIIa prior to craniotomy for evacuation of a cranial hematoma; however, the patient did not survive the trauma due to increasing intracranial pressure following craniotomy [67].

In another case report of posthip replacement bleeding following fondaparinux use, rVIIa (90 *μ*g/kg), tranexamic acid (15 mg/kg), and transfusion of packed red blood cells successfully stabilized hemoglobin levels [68]. A recent meta-analysis reported the safety of rVIIa in randomized clinical trials. Levi et al. reported higher rates of arterial events (mostly acute coronary syndrome) in patients who received rVIIa than in patients who received a placebo (5.5% versus 3.2%; OR: 1.68, 95% CI: 1.20% to 2.36%, *P* = 0.003). This difference was significant in patients ≥65 years (9% versus 3.8%; OR: 2.43, 95% CI: 1.34% to 4.41%, *P* = 0.003). The dose of rVIIa (80 to 120 *μ*g/kg) was also a significant covariate for arterial embolic events in patients who received rVIIa for central nervous system bleeding (*P* = 0.02) [69]. The cost of rVIIa use in treatment of minor-to-moderate bleeding events in patients with hemophilia has been reported to be $30,818.00 [70]. The use of rVIIa in bleeding episodes associated with fondaparinux should be done with caution given the cost, risk of thrombotic events, and partial reversibility. 

## 3. Idraparinux

Idraparinux, another long-acting factor Xa inhibitor (2.5 mg SQ weekly dose), currently is being investigated in clinical trial for the prevention and treatment of VTE. Idraparinux has a longer *t*_1/2_ (up to 60 days) and has no antidote (Table 2) [17, 18]. The van Gogh trial showed noninferiority of idraparinux versus standard of care (UFH with VKA) for VTE treatment (2.9% versus 3.0%;OR: 0.98, 95% CI: 0.63% to 1.50%). Major bleeding events in the idraparinux group versus standard-of-care group were 4.5% versus 7.0% (*P* = 0.004). In the PE arm of the study, the rate of recurrent PE in the idraparinux versus standard of care was 3.5% versus 1.6% (OR: 2.14, 95% CI: 1.21% to 3.78%), which did not meet the noninferiority criteria [71]. Recurrent VTE in six-month subgroup analysis of cancer patients enrolled in of the van Gogh trial was 2.5% in the idraparinux group compared to 6.4% in the standard of care group (hazard ration 0.39, 95%CI: 0.14–1.11). The rate of bleeding was comparable (OR 0.89, 95% CI: 0.50–1.59) [72]. These results need further investigation in forthcoming idraparinux trials. Currently, one case report of successful reversal of idraparinux with rVIIa (30 *μ*g/kg) exists in the literature [73]. The role of idraparinux in cancer patients undergoing surgical procedures also needs to be investigated. Shorter acting anticoagulants such as heparins should be used in surgical settings where epidurals are indicated.

## 4. Idrabiotaparinux

Idrabiotaparinux is another long-acting factor Xa inhibitor (3.0 mg SQ weekly dose) that has a *t*_1/2_ of up to 60 days and has no antidote (Table 2). Addition of a biotin moiety to the molecule of idraparinux has been shown in animal studies to be neutralized by avidin (egg protein), an antidote in bleeding cases. A recent study of treatment of VTE (cancer patients comprised *∼*5%) comparing idrabiotaparinux (3.0 mg SQ weekly) versus idraparinux (2.5 mg SQ weekly) for 180 days showed equal factor Xa inhibition, recurrent VTE of 2.3% versus 3.2% (difference of −0.9%, 95% CI: −3.2% to −1.4%), clinically relevant bleeding of 5.2% versus 7.3% (difference of −2.1%, 95% CI: −5.6% to −1.4%, *P* = 0.29), and a mortality rate of 1.6% versus 3.2% (difference of 1.7%, 95% CI: −3.9% to −0.5%). Three patients in the idrabiotaparinux group required avidin for planned procedures with favorable outcomes [74]. Overall results showed the comparative efficacy and safety of idrabiotaparinux to idraparinux. Further trials are needed to assess safety and efficacy of this agent in cancer patients. 

## 5. Conclusion

More targeted anticoagulants such as factor Xa inhibitors are being investigated. To date, fondaparinux has received FDA approval for the prevention and treatment of VTE. Such long-acting agents provide feasibility and better compliance than VKA or heparins. Evidence suggests that fondaparinux should be used in the acute phase of VTE treatment, but evidence of the efficacy and safety for long-term treatment is lacking, especially in cancer patients. Monitoring patients for bleeding, especially those at high risk for bleeding, is necessary owing to the fact that long-acting agent reversal is partially possible. Evidence of the newer, longer acting factor Xa inhibitors such as idraparinux and idrabiotaparinux is clearly lacking at present.

## Figures and Tables

**Table 1:** Summary of guidelines for prevention and treatment of venous thromboembolism in cancer [5–8].

Guidelines and pharmacologic prophylaxis		VKA	UFH	LMWH	FXa-I
*American College of Chest Physicians (ACCP) *		No	Yes (SQ)	Yes	Yes
(Prevention in cancer patients (medical and surgical))					

Duration of prevention: (1) For medical oncology cancer patients who have acute medical illness or who are bedridden for the duration of hospitalization. (2) For surgical cancer patients (pelvic, abdominal, orthopedic) duration of prophylaxis up to 4 weeks. (3) In the presence of contraindications or high risk of bleeding, mechanical methods may be temporarily substituted and pharmacologic prophylaxis should resume after risk of bleeding subsides.					

*American College of Chest Physician(ACCP)*	Acute	No	No	Yes	Not addressed
(Treatment in cancer patients)	Long term	Yes	No	Yes	No

Duration of treatment: At least 3 months of treatment with LMWH, followed by treatment with either LMWH or VKA.					

*American Society of Clinical Oncology (ASCO)*		No	Yes (SQ)	Yes	Yes
(Prevention in cancer patients)					

Duration of prevention: (1) For as long as the patient is hospitalized (due to surgery or acute medical illness) or until the patient is ambulatory. (2) In the presence of contraindication or high risk of bleeding, mechanical methods may be temporarily substituted and pharmacologic prophylaxis should resume after risk of bleeding subsides. (3) In certain multiple myeloma patients receiving thrombogenic chemotherapy (lenalidomide or thalidomide with dexamethasone), low-dose VKA (INR*∼*1.5) or enoxaparin (40 mg) may be considered.					

*American Society of Clinical Oncology (ASCO)*	Acute 5–10 days	No	Yes (IV)	Yes	Yes
(Treatment in cancer patients)	Long term	Yes	No	Yes	Not addressed

Duration of treatment: LMWH is preferred for 5–10 days, then LMWH for at least 6 months. VKA may be substituted if LMWH is not accessible. After 6 months of treatment, indefinite treatment duration for cancer patients with metastasis or those actively receiving chemotherapy.					

*National Comprehensive Cancer Network (NCCN)*		Yes	Yes (SQ)	Yes	Yes
(Prevention in cancer patients)					

Duration of prevention: (1) For the duration of hospitalization for medical illness and up to 4 weeks in surgical cancer patients. (2) In the presence of contraindication, use mechanical prophylaxis until bleeding risk subsides. (3) In certain high-risk medical oncology patients (i.e., aggressive tumor such as pancreatic, gastric, lymphoma, or in cases of obesity or prior VTE), longer prophylaxis is recommended. (4) In certain multiple myeloma patients receiving thrombogenic chemotherapy (lenalidomide or thalidomide with dexamethasone), VKA (INR of 2-3) or aspirin (81–325 mg) may be considered.					

*National Comprehensive Cancer Network (NCCN)*	Acute 5–10 days	No	Yes (IV)	Yes	Yes
(Treatment in cancer patients)	Long term	Yes	No	Yes	Not addressed

Duration of treatment: (1) LMWH is preferred for the first 3–6 months in DVT and 6–12 months in PE. (2) VKA can be considered if LMWH is not accessible.					

VKA: vitamin K antagonist; UHF: unfractionated heparin; LMWH: low-molecular-weight heparin; FXa-I: direct factor-Xa inhibitor; SQ: subcutaneous; IV: intravenous; INR: international normalized ratio; DTV: deep vein thrombosis.

**Table 2:** Injectable factor Xa inhibitors [17, 18].

	Fondaparinux		Idraparinux	Idrabiotaparinux
Target	Factor Xa		Factor Xa	Factor Xa
Route of administration	Subcutaneous		Subcutaneous	Subcutaneous
Prevention dose	2.5 mg		Not available	Not available
Dosing schedule	Daily		Weekly	Weekly
Therapeutic dose	Acute VTE in conjunction with VKA: (ASCO) + (NCCN) 50 kg: 5 mg once daily; 50–100 kg: 7.5 mg once daily; >100 kg: 10 mg once daily		2.5 mg	3.0 mg
Bioavailability (%)	100		100	100
Half-life (*t*_1/2_)	17 hours		60 days	60 days
Elimination	Renal		Renal	Renal
Antidote	None		None	Avidin
Market status/FDA approval	FDA approved for prevention and treatment of venous thromboembolism		In clinical trials	In clinical trials
Contraindication	(i) Patients with active bleeding (ii) Bacterial endocarditis (iii) Thrombocytopenia associated with positive antiplatelet antibodies	(i) Patients with CrCl <30 mL/min (ii) Neuraxial anesthesia (iii) Weight < 50 kg (prophylactic doses)	Not available	Not available
Monitoring parameters	Monitor hemoglobin, platelets		Not available	Not available

VTE: venous thromboembolism; VKA: vitamin K antagonist; ASCO: American Society of Clinical Oncology; NCCN: National Comprehensive Cancer Network; FDA: US Food and Drug Administration; CrCl: creatinine clearance.

**Table:** (a)

Risk factors	Odds ratio (95% CI)	*P* value	Points
Recent major bleed	2.7 (1.6–4.6)	< 0.001	2.0
Serum creatinine (Scr) > 1.2 mg/dL	2.1 (1.7–2.8)	< 0.001	1.5
Anemia	2.1 (1.7–2.7)	< 0.001	1.5
Cancer	1.7 (1.4–2.2)	< 0.001	1.0
Clinically overt pulmonary embolism	1.7 (1.4–2.2)	< 0.001	1.0
Age > 75 years	1.7 (1.3–2.1)	< 0.001	1.0

0 points = low risk.

0.1% (95% CI: 0.0–0.2).

1–4 points = intermediate risk.

2.8% (95% CI: 2.4–3.3).

>4 points= high risk.

7.3% (95% CI: 4.0–9.1).

**Table:** (b)

Risk factors	Odds ratio (95% CI)	*P* value	Points
Age > 75 years	2.16 (1.49–3.16)	< 0.001	1.0
Recent major bleed	2.64 (1.44–4.83)	0.002	1.5
Immobility ≥ 4 days	1.99 (1.40–2.83)	< 0.001	1.0
Metastatic cancer	3.80 (2.56–5.64)	< 0.001	2.0
Anemia	1.54 (1.07–2.22)	0.021	1.0
Platelet count < 100.000/mm^3^	2.23 (1.16–4.29)	0.016	1.0
Elevated prothrombin time (PT)	2.09 (1.34–3.26)	0.001	1.0
Creatinine clearance (CrCl) < 30 mL/min	2.27 (1.49–3.44)	< 0.001	1.0
Distal deep vein thrombosis	0.39 (0.16–0.95)	0.038	−1.0

Score < 1.5 = low risk 0.16%.

LR = 0.29 (95% CI: 0.20–0.41).

Score 1.5–4 = intermediate risk 1.06%.

LR = 1.92 (95% CI: 1.69–2.17).

Score > 4 = high risk 4.24%.

LR = 7.95 (95% CI: 5.42–11.6).
